# Comparative Study of [^18^F]DPA714 and [^18^F]FDG PET Tracers in an Experimental Model of Pulmonary Tuberculosis

**DOI:** 10.1007/s11307-025-02057-6

**Published:** 2025-10-21

**Authors:** M. A. Stammes, M. P. M. Vierboom, C. C. Sombroek, J. Bakker, L. Meijer, R. A. W. Vervenne, S. O. Hofman, E. Nutma, I. Kondova, A. D. Windhorst, J. A. M. Langermans, F. A. W. Verreck

**Affiliations:** 1 Biomedical Primate Research Centre (BPRC), Lange Kleiweg 161, Rijswijk, 2288GJ the Netherlands; 2 Department of Radiology and Nuclear Medicine, Tracer Center Amsterdam (TCA), Amsterdam University Medical Centre, Vrije Universiteit, Amsterdam, the Netherlands; 3 Department of Population Health Sciences, Faculty of Veterinary Medicine, Unit Animals in Science and Society, Utrecht University, Utrecht, the Netherlands

**Keywords:** PET-CT, Non-human primates, Macaques, TB, TSPO, Lungs

## Abstract

**Purpose:**

Tuberculosis (TB) continues to afflict global health. Therefore, a deeper understanding of the host response mechanisms that underly pathogenesis versus disease control upon infection with *Mycobacterium tuberculosis* (*Mtb*) is required to leverage the development of improved therapeutic or prophylactic TB treatment regimens. In the present work positron emission tomography (PET) using [^18^F]DPA714 is piloted as a tracer of the mitochondrial translocator protein TSPO that mainly targets macrophages.

**Procedures:**

We compared two tracers: [^18^F]DPA714 to the widely applied marker [^18^F]FDG to visualize the development of experimental pulmonary TB in three rhesus monkeys (*Macaca mulatta*), that were infected with *Mtb* by repeated low dose exposure. Next to baseline recordings prior to infectious challenge, two PETs at a two-weeks interval were acquired early after the manifestation of TB infection for each of the respective tracers.

**Results & Conclusions:**

Here, we demonstrate that both PET tracers detected *Mtb* infection. The inflammatory response tracked by [^18^F]FDG progressively increased in line with the developing TB pathology, while [^18^F]DPA714 showed a transient signal in lungs and lung-draining hilar lymph nodes. This study underpins the potential value of different tracers to investigate cellular and molecular host response cascades in experimental medicine settings, in this case, into a (transient) local involvement of myeloid immune cell activation versus inflammation-associated glucose consumption in pulmonary TB.

**Supplementary Information:**

The online version contains supplementary material available at 10.1007/s11307-025-02057-6.

## Introduction

Tuberculosis (TB), mostly resulting from pulmonary *Mycobacterium tuberculosis* (*Mtb*) infection, continues to be a leading cause of mortality and morbidity in the world [1]. To effectively push back on the ongoing epidemic, better tools to improve prophylactic and therapeutic regimens as well as our diagnostic capabilities should be developed [2]. However, the swift development of improved TB treatments and vaccines is hampered by an incomplete understanding of the host response cascades that underly pathogenesis versus protective immunity. Molecular imaging modalities in combination with dedicated tracers as diagnostic tools, provide the potential to investigate the biology of *Mtb* infection by visualising cellular and molecular host response dynamics towards a better understanding of TB pathophysiology. Moreover, non-invasive imaging of TB can enhance the development of new therapies and vaccines by accelerated or refined readout of treatment efficacy both clinically and preclinically [3–6].

Over the last decades, an array of molecular imaging protocols based on positron emission tomography (PET), or single photon emission computed tomography (SPECT), have been developed establishing themselves as highly sensitive assets in the diagnostic toolbox [3, 5, 7]. Both PET and SPECT rely on radiolabelled tracers with specific affinity for molecular or cellular markers of physiological processes linked to disease. These molecular imaging platforms have already been exploited successfully to monitor TB [3–6, 8, 9].

The most intensively investigated and widely applied PET tracer so far, also in TB research and diagnostics, is the glucose derivative 2-[^18^F]deoxy-2-fluoro-D-glucose ([^18^F]FDG) [4–6, 10]. FDG, upon injection, gets trapped in cells with a high metabolic turnover which is, among others, prominently associated with inflammation. The inflammatory response in TB is particularly evident in granulomatous lesions that occur in the lungs as a hallmark of pulmonary *Mtb* infection [5]. These TB lung granulomas are differentiated pathologic structures of various host immune cells. The granulomas are characterised by primary myeloid macrophages providing a major niche of persistent intracellular *Mtb* infection, surrounded by secondary lymphoid T and B cells. TB granulomas reflect the response of the host trying to eliminate and/or contain the infection. [^18^F]FDG-PET displays a high sensitivity to measure inflammation and in combination with computed tomography (CT) registering anatomical changes in lungs as a result of TB pathology.

However, the specificity of [^18^F]FDG-PET is limited, since glucose consumption can be upregulated under various pathologic conditions as well as under sterile conditions of increased cell metabolism like host immune activation. Therefore, there is an urge to develop and/or investigate other tracers that may not be more specific per se but will allow to broaden the range of molecular medical imaging towards differentiating distinct processes underlying TB pathogenesis. One such alternative PET imaging strategy that may be of use for TB research is targeting the translocator protein (TSPO). TSPO is an outer mitochondrial membrane receptor that is particularly targeted in neuroscience where it is mostly present in microglia and astrocytes, the innate immune cells of the central nervous system, under conditions of (autoimmune) inflammation [11–16]. However, TSPO expression is not limited to the immune cells of the central nervous system but it is also prominent in activated myeloid (rather than lymphoid) immune cell populations throughout the body [11–16]. Over the last years over twenty different TSPO targeting PET tracers have been developed, many of which are in different phases of development and display their own specific strengths and limitations. Notably, [^18^F]DPA714, as tested in rodents, NHPs and humans, has a suitable lipophilicity and appears effective in visualizing neurological disorders [17, 18].

In a preclinical C3HeB/FeJ mouse TB model, tracing TSPO by SPECT with [^125^I]DPA713 enabled the identification of tuberculous lesions, while the same tracer also appeared a useful biomarker of antibiotic treatment and bactericidal activity [19]. Exploring rabbits as a model of paediatric TB, increased TSPO-targeted PET signals of [^124^I]DPA713 were reported in the white matter of the brain and around TB lesions as signs of central nervous system involvement [20]. In the translational perspective, however, and to our best knowledge, the value of TSPO for imaging TB in the human or the non-human primate (NHP) host remains to be determined [16].

While we have previously shown that the TSPO tracer [^18^F]DPA714 enables visualisation of pulmonary myeloid cell activation as a result of experimental SARS-CoV2 infection in rhesus macaques [15], we set out in the present study to investigate if this tracer can detect experimental *Mtb* infection. To this end, we compared [^18^F]DPA714-PET with [^18^F]FDG-PET imaging at early stages of pulmonary *Mtb* infection in a pilot setting. Since experimental infection of rhesus macaques with virulent M.tb strain Erdman typically induces acute progressive TB in untreated animals, we here focus on early time points and report on the differential dynamic of [^18^F]DPA714- versus [^18^F]FDG-PET signals, extensively involving lungs and lung-draining hilar lymph nodes (LNs). Transient [^18^F]DPA714-PET signals were observed, compatible with an impermanent influx of activated myeloid cells/macrophages to the site of infection in lungs and hilar LNs, while the inflammatory response increases progressively. Thus, the data show that targeting TSPO via [^18^F]DPA714 by PET can detect (early) TB in NHPs and underpin that different PET tracers have the potential to delineate distinct processes underlying TB pathology.

## Materials and Methods

### Ethics & Animals

This study was performed in three male rhesus macaques (*Macaca mulatta*) at the Biomedical Primate Research Centre (BPRC, Rijswijk, Netherlands) under project license AVD5020020209404 which was issued by the competent national authorities (Central Committee for Animal Experiments). The animals were socially housed, in pairs, during the study. All possible precautions were taken to ensure the welfare and to avoid any discomfort to the animals.

Selected animals were negative for prior exposure to mycobacteria and were challenged with *Mtb* strain Erdman K01 (BEI Resource, VA, USA) following a repeated low dose protocol as described before [21]. For additional information see supplementary material.

### IFNγ ELISpot Assay on Peripheral Blood Mononuclear Cells (PBMC)

An NHP-specific IFNγ ELISpot assay was used on PBMC, according to manufacturer’s protocol (U-CyTech, Utrecht, Netherlands), to determine the frequency of antigen-specific IFNγ producing cells on a weekly basis. In brief, 200,000 freshly isolated PBMC were incubated in triplicate for 24 h with specific PPD antigen or culture medium as a control. Subsequently, supernatant was collected and stored (− 80 °C), and cells were transferred to specific anti-IFNγ coated filter plates (PVDF, Millipore) for an additional overnight (18 h) incubation. Cells were discarded and membrane-bound IFNγ was detected using biotinylated anti-IFNγ antibody, streptavidin–horseradish peroxidase conjugate, and tetramethylbenzidine (TMB) substrate (MAbTech, Stockholm). Spots were quantified using an automated AID ELISpot Reader (Autoimmune Diagnostika GmbH, Strassberg) and are reported as antigen specific signals after control correction.

### Radiotracers

[^18^F]FDG was purchased from a commercial provider (GE Healthcare, Leiderdorp, Netherlands). Radiosynthesis of [^18^F]DPA714 was performed using procedures and in-house constructed automatic devices as described previously [12]. [^18^F]DPA714 was produced with an average molar activity of 40.5 GBq/*μ*mol (range 30.7—57.9 GBq/*μ*mol on activity reference time), a radioactivity concentration of 212 MBq/ml, and a radiochemical purity of at least 98.0%.

### PET-CT Acquisition, Reconstruction, and Analysis

**Fig. 1:**
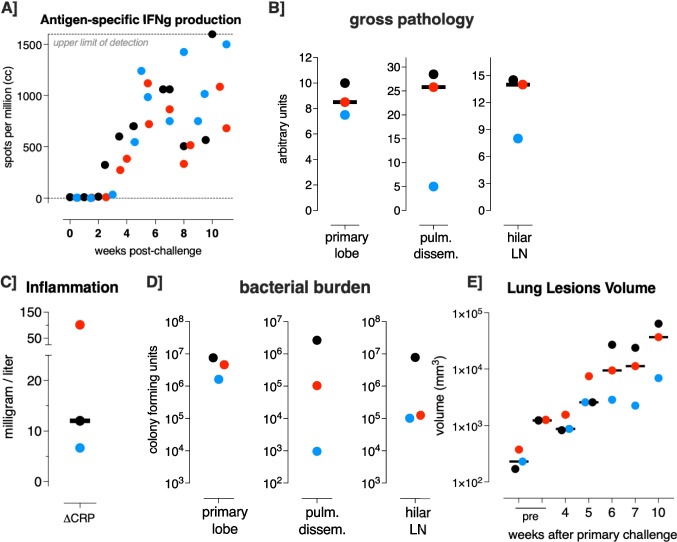
Longitudinal Disease Monitoring. Experimental *Mtb* infection and TB disease were verified by standard readouts of specific host responses, based on immunology, pathology and bacteriology. **A** Along the *Mtb* challenge phase infection was confirmed by an increasing frequency of antigen-specific IFNγ-secreting cells in the circulation from week 3 after the first exposure onwards. Individual control-corrected data are plotted. **B-D** At study endpoint *postmortem* evaluation confirmed pulmonary TB disease and *Mtb* outgrowth in all three animals. **B** Pathological involvement is scored in arbitrary units (from left to right) for the primary targeted lung lobe, the other lung lobes summed together as a measure of pulmonary dissemination of disease, and the lung-draining hilar lymph nodes (LN). Individual data are plotted, with horizontal lines indicating medians. **C** C-reactive protein (CRP) levels in serum were elevated, presenting an acute phase response. **D** Mycobacterial burden was enumerated as colony forming units from serial dilutions of tissue homogenates, with data represented as for pathology in [B].** E** Over time the volume of the lung lesions is based on discrimination of Houndsfield Units on CT. These values are independent of the PET tracers used and can, thus, be directly compared. Individual data are plotted, with bars indicating medians

A PET-CT was acquired pre-infection for both [^18^F]FDG and [^18^F]DPA714 to obtain baseline values. Weekly PET-CTs were obtained starting with the first scan four weeks after the first of repeated limiting dose challenges (Fig. 1), using alternatingly [^18^F]DPA714 and [^18^F]FDG as a tracer. The scans were obtained with a MultiScan Large Field of View Extreme Resolution Research Imager (LFER) 150 PET-CT (Mediso Medical Imaging Systems Ltd., Budapest, Hungary) as described before [22]. Animals were fasted overnight and sedated with ketamine (10 mg/kg ketamine hydrochloride; Alfasan Nederland BV, Woerden, Netherlands) combined with medetomidine hydrochloride (0.05 mg/kg; Sedastart; AST Farma B.V., Oudewater, Netherlands), both administered intramuscularly. The animals were intubated but not mechanically ventilated, maintaining anaesthesia with isoflurane (1.5–2.0%) during the entire scan procedure. The animals were positioned head-first supine (HFS) with the arms up. After the scan, upon return to their home cage, atipamezole hydrochloride (5 mg/ml, 0.25 mg/kg; Sedastop, ASTFarma B.V., Oudewater, Netherlands) was administered intramuscularly to antagonize medetomidine. After an intravenous bolus injection of [^18^F]FDG (± 100 MBq in 4 ml) or [^18^F]DPA714 (± 180 MBq in 1 ml) and an incubation time of 45 min ([^18^F]FDG) or 30 min ([^18^F]DPA714), a PET image of a single field-of-view covering the chest area was obtained for 15 min. The incubation time for [^18^F]DPA714 was defined by experience from former NHP experiments [15, 23].

During incubation a chest CT was obtained which was retrospectively gated in the expiration phase [15]. The CT was captured both for diagnostic evaluation and fusion with the PET as described previously [24]. The emission data were iteratively reconstructed (OSEM3D, 8 iterations and 9 subsets with an isotropic voxel size of 0.8 mm) into a single frame PET image normalized and corrected for attenuation, scatter, random coincidences using the free-breath CT, and radioactive decay. The analysis was performed with VivoQuant 4.5 (Invicro, Boston, USA).

For the quantitation, standard uptake values (SUV) were determined [25]. We discriminated the following 3D-generated volumes of interest (VOIs): anatomically unaffected lung tissue, lung lesions, and LNs. For the lungs the parameters SUVmean, SUVpeak and SUVsum were determined. The SUVmean is calculated as the average intensity within a ROI. The SUVpeak represents the average activity of a defined volume (1 mm^3^) in the peak area, and thus, is affected less by noise than the SUVmax [22]. The SUVsum is the sum of the average activity of all voxels within a ROI. These three measures enable to differentially describe the respective aspects of the PET signals.

The procedure to define the different VOIs consists of several steps (Supplemental Fig. 3). First, healthy lung tissue was determined based on a density range (from −1000 to −500 HU) and based on this the PET signals associated with pulmonary lesions were quantified. The definition of the LNs was based on a lower threshold standard uptake value (SUV) of 1.0 to discriminate from the surrounding tissue. This value is based on previous studies and after a visual check in the scans obtained at the last imaging timepoint. However, as the animals are followed longitudinally, not the threshold-value per se but applying the same threshold for all timepoints is critical for registering longitudinal changes. Afterwards the LNs were quantified using the same parameters as the lung lesions, now in combination with two additional parameters; the volume and the anatomical density, represented as the average Hounsfield Unit (HU) measured. These two parameters were solely based on CT but not independent from the PET tracer that was used, since a SUV threshold was applied for the definition of the LN VOIs.

### Immunohistochemistry

Paraffin sections of 5 µm were de-paraffinized using xylene, rehydrated by passaging through alcohol solutions of descending concentration, and washed in phosphate buffered saline. Endogenous peroxidase activity was blocked using 0.3 (w/v) H_2_O_2_. Following subsequent washing, paraffin sections underwent antigen retrieval in citrate buffer (pH 6.0) in a water bath at 95 °C for 30 min. After cooling, sections were washed and incubated overnight in primary antibodies diluted in universal antibody diluent (U3510, Sigma Aldrich) containing bovine serum albumin (BSA). After subsequent washing, sections were incubated with poly-horseradish peroxidase (HRP) labelled secondary antibodies (K5007, Agilent) for 45 min, washed, and developed for visualisation with 3,3'-diaminobenzidine (DAB, K5007, Agilent) for 5 min after which sections were rinsed with tap water to fix the chromogen. Slides were counterstained with haematoxylin, dehydrated with ascending alcohol solutions and xylene and mounted with malinol (Waldeck ref 3C-242). Eventually, the slides were examined by microscopy to localize the proteins of interest: TSPO (ab109497, Abcam, EPR5384, 1:750), CD3 (A0452, Agilent, Polyclonal, 1:500), CD68 (M0814, Agilent, KP1, 1:200). Appropriate negative controls were used by omitting the primary antibodies.

## Results

To assess the potential of mitochondrial TSPO as a molecular target for PET-mediated diagnosis of TB infection and disease, three adult rhesus macaques were experimentally infected with a virulent strain of *Mtb* (strain Erdman). Infection was established by weekly exposure to a single colony forming unit (CFU) of *Mtb* through endobronchial instillation into the lower left lung lobe for 8 consecutive weeks.

Establishment of TB infection was confirmed by standard readouts including post-mortem evaluation. In line with previous findings in this infection model [21], a primary immune response against *Mtb* became evident from 3 to 4 weeks after the first challenge by showing increased frequencies of antigen-specific IFNγ-secreting peripheral blood mononuclear cells (PBMC) (Fig. 1A). At endpoint, post-mortem assessments demonstrated that (primary) TB, as expected, was apparent in the endobronchial targeted lower left lung lobe in all three animals, showing near maximum scores by a predefined scoring algorithm [26] (Fig. 1B). Two out of three animals also showed extensive pulmonary dissemination to other lung areas, while also their lung-draining hilar LNs were most severely affected (Fig. 1B). This was consistent with what was found at the last PET-CT timepoint for both the lung lobes and LNs. These two animals concordantly presented with extra-thoracic disease involving spleen and/or liver (not shown) and presented an acute phase response by elevated C-reactive protein (CRP) levels in serum at endpoint (Fig. 1C). Mycobacterial outgrowth was demonstrated in all by the recovery of considerable counts of CFU from primary and secondary lung lobes as well as from the hilar LNs (Fig. 1D).

To assess the development of disease in the lungs and lung-draining hilar LNs in vivo over time, PET-CTs were obtained at multiple timepoints. The area of affected lung measured by CT was increased over time (Fig. 1E) [22]. As a hallmark of experimental TB in this species of highly susceptible (naive) rhesus macaques, prominent hilar LN involvement was corroborated by increased PET tracer uptake. (Fig. 3). Thus, both lung lesion volume and hilar LN involvement confirmed a progressive active disease manifestation. While volume definition of the lungs and lung lesions is independent of the PET tracer applied, we found CT density and average PET tracer uptake of anatomically unaffected lung tissue constant over the entire course of the experiment for both tracers (Suppl. Figure 2), thus underpinning the robustness of our lung lesion definition over time.

**Fig. 2:**
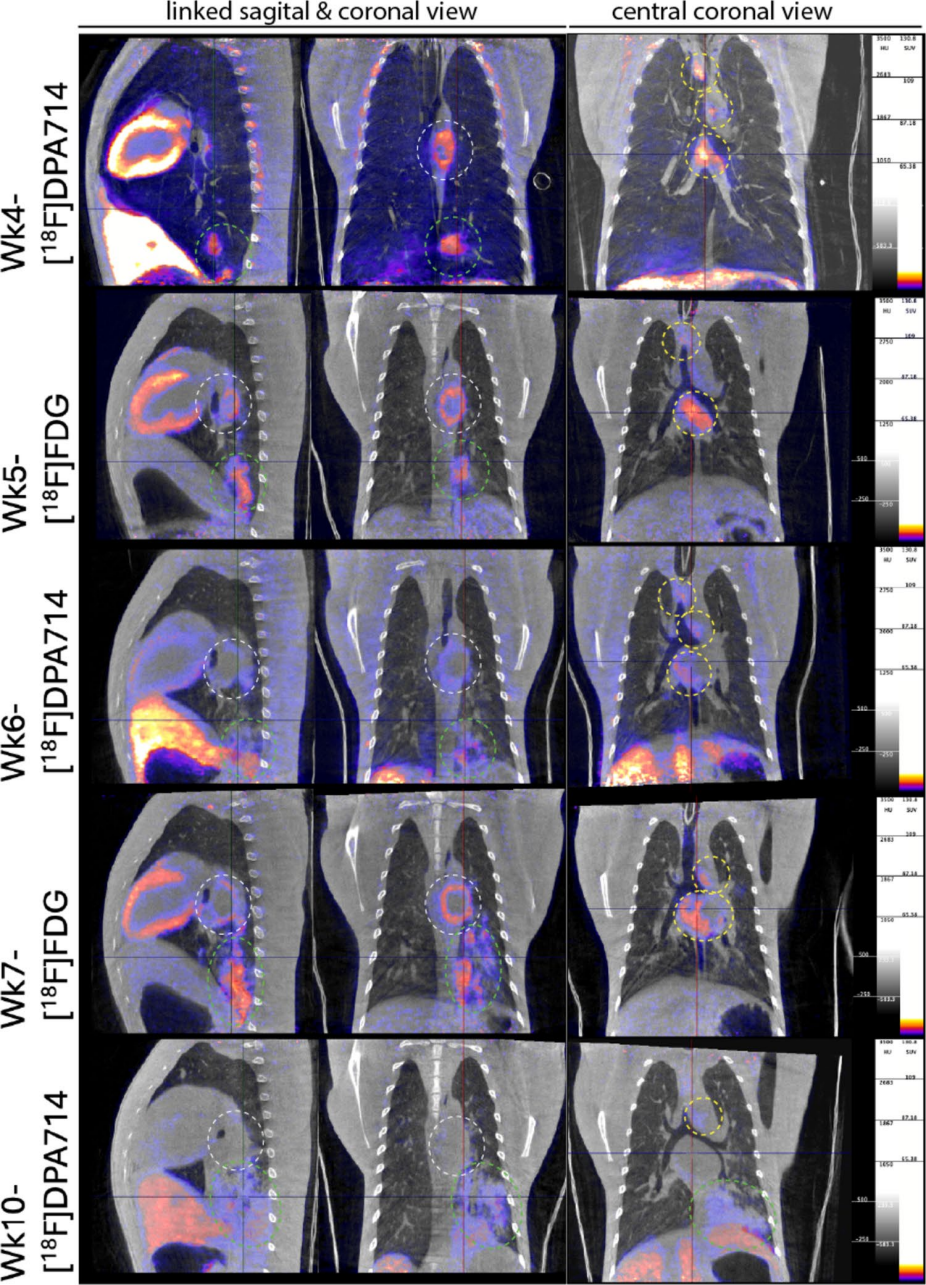
Overview of both the pulmonary lesions and hilar lymph nodes on the PET-CT with [^18^F]FDG and [^18^F]DPA714 (TSPO) over time of one of the three animals (of the animal represented by the red dots in the graphs). The left and middle column are linked images, the crosshair visible in the centre of the images represents the same spot, with the same representation of the sagittal and coronal view over time. The right column visualises the central coronal view throughout the different scans. The green and white dotted circles represent the same lesion over time, the green circles mark the lung lesion, which is growing in all directions, the white circle shows a hilar lymph node which develops a necrotic core over time, visualised by the lack of signal from the centre of the lymph node. This development of necrosis is not apparent in the lung lesions. The yellow circles represent different lymph nodes than the one marked with the white circle

**Fig. 3:**
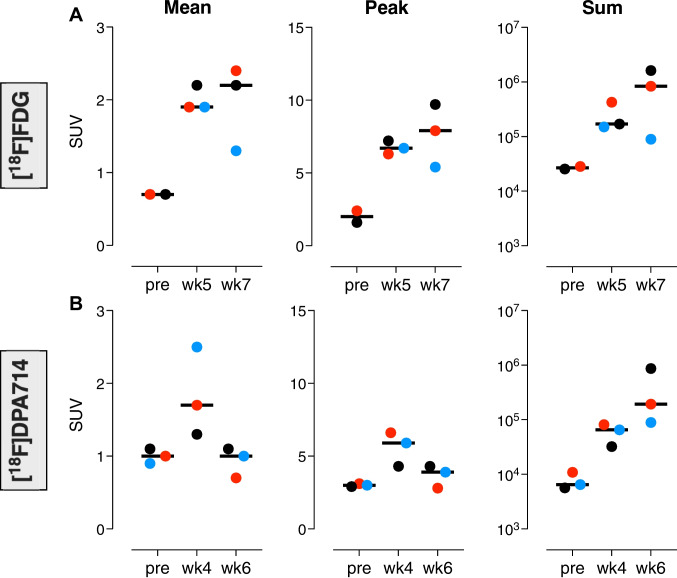
Quantitative analysis of the pulmonary lesions of the PET signals with [^18^F]FDG and [^18^F]DPA714 (TSPO) over time. The PET signals are quantified based on the SUVmean, SUVpeak and SUVsum parameters over time. The lung lesion VOI is defined as the lungs minus the anatomically unaffected lung tissue based on differences detected in Hounsfield Units on the CT. This makes the definition of this VOI independent of the tracer used. FDG baseline scan for n = 2 only due to technical error. **A** represents the data obtained with the use of [^18^F]FDG while (**B**) is based on [^18^F]DPA714 TSPO PET-signals. Individual data are plotted, with bars indicating medians

By the time diagnostic IGRA test conversion had confirmed infection in either of these animals (see above, Fig. 1A), the first images after infectious challenge with [^18^F]DPA714 (at week 4) as well as [^18^F]FDG (at week 5) yielded elevated PET signals both by qualitative and quantitative analyses with the various PET read-out parameters in the lungs: SUVmean, SUVpeak and SUVsum (Figs. 2 & 3). Both tracers detected primary lesions in the bronchoscope-targeted lower left lung lobe (see Fig. 3).

Over time, based on the CT, these lesions were increasing in size, as expected in this rhesus macaque TB model. Interestingly, however, lesion-associated SUVmean and SUVpeak of the [^18^F]DPA714-PET (Fig. 3B) decreased over a two-weeks follow up, while those signals for [^18^F]FDG (Fig. 3A) continued to increase over a similar two-weeks interval (with an off-set of 1 week relative to the [^18^F]DPA714-PET recordings (Suppl. Figure 1). This differential kinetic in SUVmean and SUVpeak, however, did not appear from comparing the developing SUVsum signals, which showed an increasing pattern for both tracers. The latter is likely explained by a sharper increase in lesion volume relative to the decrease in SUVmean of the [^18^F]DPA714-PET signal, as a result of which the SUVsum signal over that two-weeks interval still increases. An alternative explanation based on early formation of necrotic lesions can be partly ruled out for the lung lesions but not for the lymph nodes by the fact that the [^18^F]FDG signals over the same time period with a one week delay, relative to the [^18^F]DPA714) scan, showed a continuously increasing SUV.

**Fig. 4:**
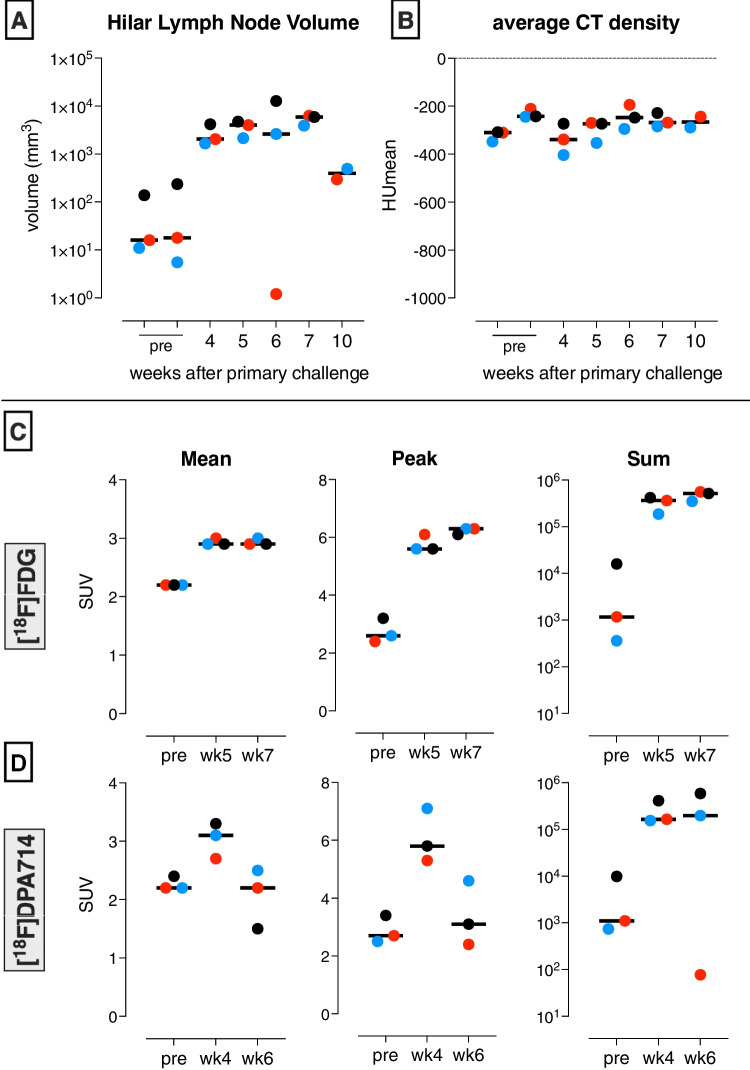
Quantitative analysis of the lung-draining hilar lymph nodes based on both the CT and PET signals with [^18^F]FDG and [^18^F]DPA714 (TSPO) over time. The lymph node VOIs are defined based on a SUV threshold of 1.0. The lymph nodes are quantified both on CT (**A-B**) and PET (**C-D**) parameters over time. As the CT values are based on a PET threshold, the results are not independent of the PET tracer applied. Individual data are plotted, with bars indicating medians

The HUmean of the LNs remained constant over time (Fig. 4A), indicating that the density of the VOI which defines the LN based on PET signals did not change, while their size increased over the respective time interval (Fig. 4A). For the PET-CT analysis of the lung-draining hilar LNs a similar longitudinal pattern was detected as for the lung lesions. By [^18^F]FDG scanning both the volume of LNs and the SUV parameters showed increasing signals over time (Fig. 4B), whereas by [^18^F]DPA714 the PET signals declined again from week 4 onwards (Fig. 4C). In contrast to the lung lesions, for the LNs formation of necrosis was detected by a reduced PET signal from the centre of the lesions, also confirmed during necropsy. In addition, for the LNs also the SUVsum decreased towards the last timepoint, suggesting a transient presence of activated macrophages.

**Fig. 5:**
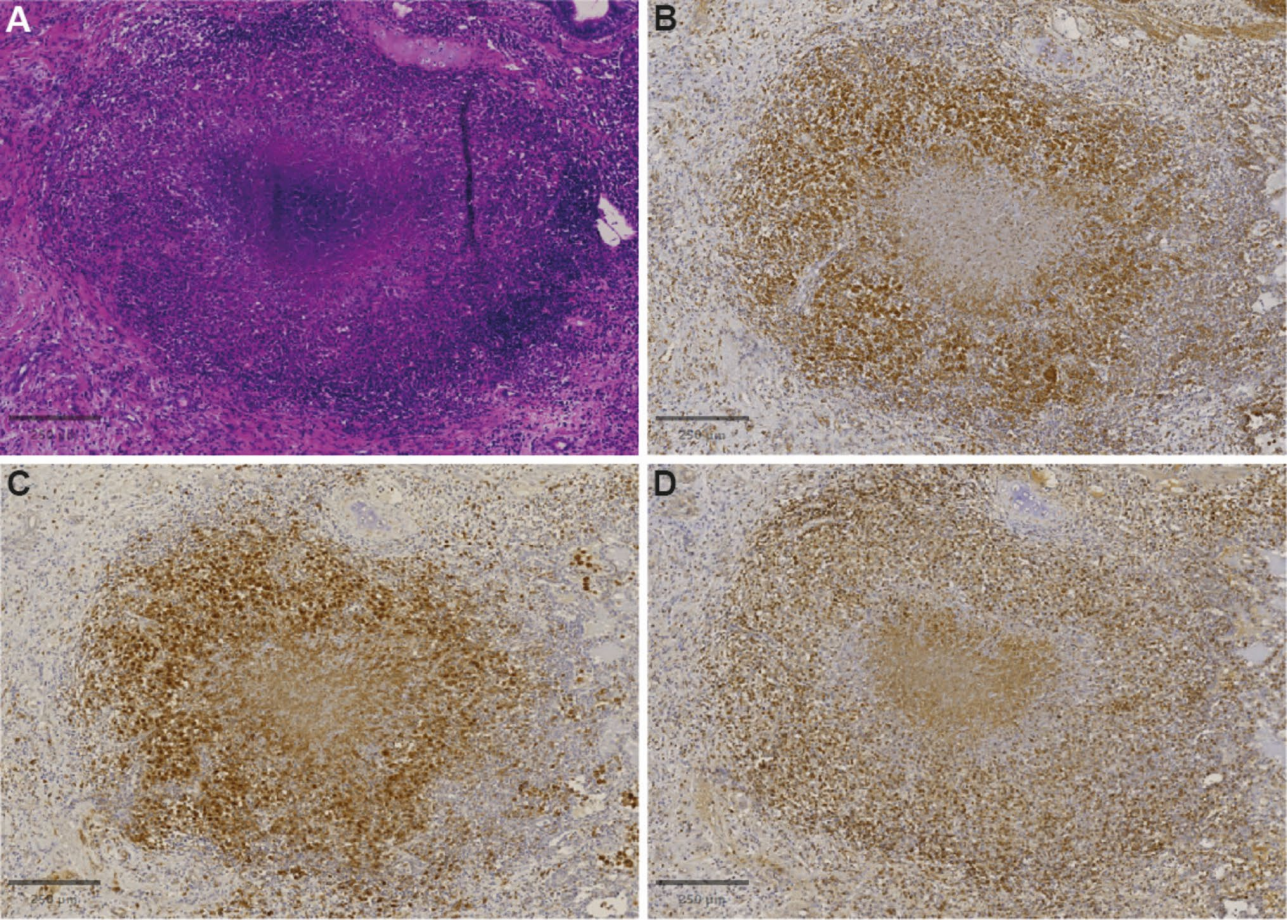
Photomicrograph of a pulmonary TB granuloma. **A** HE staining showing a central area of necrosis with large numbers of viable and degenerate neutrophils mixed with eosinophilic cellular and karyorrhectic debris. The necrotic core is surrounded by numerous epithelioid macrophages and occasional multinucleated giant cells and peripherally rimmed by lymphocytes and plasma cells. **B** TSPO immunohistochemistry (IHC) staining appears around the necrotic centre. **C** CD3 IHC-staining defines the rim of the T cells in the periphery (**D**) CD68 IHC-staining shows the epithelioid macrophages within the granuloma. (solid lines represent identical scale bars of 250 μm)

To further substantiate the potential of [^18^F]DPA714 to track lesion formation and potentially the presence of myeloid cells during the TB infection, we evaluated formalin-fixed paraffin-embedded (FFPE) lung tissue collected at study endpoint by immunohistochemistry. Antibodies were selected for specific staining of TSPO as the target of [^18^F]DPA714, as well as CD3 and CD68 as T lymphocyte and macrophage markers, respectively. By immunohistochemistry, TSPO staining appeared in association with TB granulomas (and bronchial epithelium; not shown), demonstrating that TSPO expression in pulmonary TB coincides with the occurrence of granulomatous lesions (Fig. 5).

## Discussion

Molecular imaging by PET-CT provides a powerful, non-invasive diagnostic platform for investigating infectious diseases, including TB following from *Mtb* infection [27, 28]. A recent review stated that PET-CT has the potential to revolutionize research in TB to follow the spatial distribution of the disease [6]. The value of PET-CT as read-out parameter to better understand host–pathogen interactions is highly dependent on the radiotracer applied. [^18^F]FDG is the most common tracer used with a high sensitivity though with limited specificity.

In the present study, designed as a pilot to provide *proof of concept* in the NHP host, we traced early *Mtb* infection through the mitochondrial translocator protein TSPO associated with macrophage activation, using [^18^F]DPA714. In a comparative approach in rhesus macaques, we demonstrated that [^18^F]DPA714, like [^18^F]FDG, detected early granulomas and lung-draining lymph node involvement upon experimental low dose *Mtb* infection but, unlike [^18^F]FDG, showed a differential dynamic. While within 2 months from initial endobronchial *Mtb* exposure [^18^F]FDG signals continuously increased, [^18^F]DPA714 rather showed a transient elevation of mean and peak signals. This finding is in line with the differential detection of distinctive processes underlying TB pathogenesis, in which [^18^F]FDG traces an increasing inflammation-associated glucose consumption reminiscent of acute progressive TB disease, whereas [^18^F]DPA714 tracks an early macrophage response that appears locally transient.

This work, to our best knowledge, reports a ‘first in NHP’ finding, while previously Foss et al. [19] have already demonstrated the successful targeting of TSPO by molecular imaging for detecting experimental *Mtb* infection in mice [19]. In mice, lesion-specific signal-to-noise ratios of a TSPO tracer were two times higher than that of [^18^F]FDG. This enhanced sensitivity of TSPO, however, was not apparent from our NHP data, which may be explained by model specific characteristics (e.g. TSPO regulation, body size or experimental infection dose), or using SPECT versus PET. Also, the mouse study reported increased TSPO signals post infection in anatomically unaffected lung tissue, which was not detectable in our TB study in NHP. However, we have previously reported a similar elevation of PET signals for TSPO in healthy lung parenchyma when investigating [^18^F]DPA714 images of SARS-CoV-2 infection in rhesus macaques [15]. While Foss et al. [19] considered it a limitation to the specificity of the tracer, it seems plausible that – depending on species, pathogen and/or the experimental infection condition—elevated TSPO-specific PET in anatomically unaffected lung is indicating a biologically pertinent involvement of activated macrophages as part of an organ-specific infection response that may be independent of lesion formation per se.

Interestingly, our observation of decreasing [^18^F]DPA714 towards later timepoints and against a progressive TB disease development, was similarly noticed in an Ebola virus (EBOV) infection study in NHPs [29]. In this study it was demonstrated that macrophages, monocytes and dendritic cells display highest TSPO expression levels per se, while the [^18^F]DPA714 PET signal of TSPO went down in lung and spleen but not in bone marrow within days of EBOV infection. The authors suggested that the [^18^F]DPA714 PET signal truly reflects the collective status of multiple immune cell populations, implicating emergency haematopoiesis in bone marrow and progressive monocytic and lymphocytic depletion in spleen. They hinted that the decreasing lung signal might be due to a depletion of alveolar macrophages. Such an effect, however, seems less likely in the present case of experimental TB, while it has been shown that alveolar macrophages are the primary target of infection but swiftly translocate and disseminate the infection to other myeloid cells in the interstitium, including monocyte-derived antigen-presenting cells [30, 31]). As an alternative to specific cell subset expansion and/or contraction, functional macrophage plasticity and polarisation over the infection follow-up time should be considered [32]. Although type-2 anti-inflammatory macrophages appear associated with TB disease progression [33, 34], paradoxically TSPO was found down-regulated in pro-inflammatory type-1 macrophages [35]. Our pilot setup unfortunately leaves these points unresolved. However, the present study warrants further investigation to understand the dynamic of [^18^F]DPA714 PET signals in relation to initial and sustained innate TB immunity as well as the developing adaptive response on local macrophage biology in the course of experimental pulmonary *Mtb* infection.

TSPO is primarily located in the outer membrane of mitochondria, disputably overexpressed in activated microglia and astrocytes or vascular endothelial cells during neuroinflammation, and detectable by high affinity binding of DPA derivatives labelled for PET (or SPECT) tracing. Thus, while TSPO imaging so far has most extensively been exploited in neurobiology, our data and the aforementioned publications indicate that TSPO imaging could also be utilised in infectious diseases where it most likely reflects activation and/or infiltration of mononuclear phagocytes/macrophages [13, 15, 36].

In humans, it has been shown that PET tracer affinity for TSPO ligands can be affected by the rs6971 polymorphism [37, 38]. While we have no evidence of such TSPO polymorphism in NHPs yet [13, 36], in experimental settings like the present study, longitudinal PET imaging with appropriate individual baseline recording practically overcomes this caveat. For humans the same is true when scans of one patient can be longitudinally compared towards each other. As current TB immune diagnostics without longitudinal record critically fail to discriminate early infection from later-stage/remote asymptomatic manifestation, it is tempting to speculate that combined use of differential PET tracers, like [^18^F]DPA714 versus [^18^F]FDG, could potentially enhance the differential diagnosis of *M.tb* infection stages, at least in clinical experimental and/or limited trial settings.

Despite scientific progress, many details of the pathophysiological response underlying TB disease or protective immunity against *Mtb* infection remain elusive. In tractable modelling of TB infection and disease molecular imaging like PET-CT can provide new information and insight into the underlying molecular and cellular cascades by using different tracers with diverse specificity. Our results presented here demonstrate that both [^18^F]FDG and [^18^F]DPA714 can be used to visualize early TB infection and disease, with the latter only showing transiently elevated signals. Since macrophages are classically the primary target of *Mtb* infection yet functionally rather dynamic and involved in both pro-inflammatory immunity as well as homeostatic control [39, 40], finding a differential PET signal of activated macrophages in the first 4 to 6 weeks after infectious challenge warrants future investigation to learn if this specific [^18^F]DPA714 PET tracer can be meaningfully added as a diagnostic in research supporting the development of better tools to fight TB.

## Supplementary Information

Below is the link to the electronic supplementary material.

ESM1 (DOCX 2.22 MB)

## Data Availability

Data will be available upon request.
