# Supplementary material for: Comparative Study of [18F]DPA714 and [18F]FDG PET Tracers in an Experimental Model of Pulmonary Tuberculosis
Source: Mol Imaging Biol. 2025 Oct 21;27(6):943–53. doi: 10.1007/s11307-025-02057-6 (PMC12804315; doi:10.1007/s11307-025-02057-6)
Supplement: Supplementary file 1 — (DOCX 2.22 MB) [file 11307_2025_2057_MOESM1_ESM.docx]

**Supplemental Material**

**Comparative study of [^18^F]DPA714 and [^18^F]FDG PET tracers in an Experimental Model of Pulmonary Tuberculosis**

**Material & Methods**

Ethics

This study was performed at the Biomedical Primate Research Centre (BPRC, Rijswijk, Netherlands) under project license AVD5020020172645, which was issued by the competent national authority (Central Committee for Animal Experiments). Further approval was obtained after assessment of the study protocol by the institutional animal welfare body. All procedures, husbandry, and housing were performed in accordance with Dutch law on animal experimentation and the EU Directive 63/2010. The BPRC is accredited by the American Association for Accreditation of Laboratory Animal Care (AAALAC) International.

Animals

Three healthy purpose bred Indian-origin male rhesus monkeys (*Macaca mulatta*; age 4 y, weight range 6.0-8.2 kg) were included in this study. Weight-to-Height index (WHI) is added as body-mass parameter providing information regarding body composition in relation to image quality. To preserve image quality a WHI pre-selection criteria was set of 42-67 kg/m^2.7^ for rhesus macaques [21-22]. Animals were declared healthy based on a complete physical, haematological, and biochemical evaluation performed before inclusion. Animal characteristics are listed in Supplementary Table 1 and a schematic diagram of the study design is depicted in Supplementary Figure 1.

**Supplemental Table 1: Animal Characteristics**

| ID | Gender | Age (yrs) | Body Weight (kg) | WHI (kg/m^3^) |
| --- | --- | --- | --- | --- |
| 1 | Male | 4.6 | 7.2 | 47.6 |
| 2 | Male | 4.7 | 8.2 | 51.4 |
| 3 | Male | 4.7 | 6.0 | 49.8 |

Three healthy, young adult, male rhesus macaques were selected for a TB infection experiment. Age, body weight, and the Weight-to-Height index (WHI) are listed.


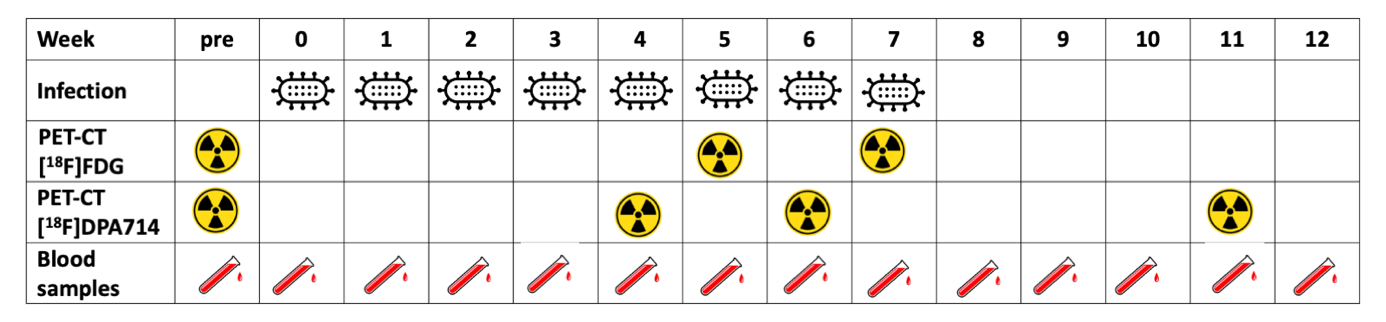


**Supplemental Figure 1: Schematic overview of the experimental procedures, related to this manuscript.**

Animals were challenged by exposure to *M. tuberculosis (Mtb)* in a *repeated limiting dose* (RLD) infection protocol, based on weekly endobronchial instillation of a single colony forming unit (CFU) for 8 consecutive weeks. Blood was collected weekly for diagnostic host response analysis by antigen-specific interferon-gamma release. PET-CTs were performed at indicated timepoints, using alternatingly [^18^F]FDG or [^18^F]DPA714. For the last imaging timepoint a CT of the lungs was obtained from all animals but only from one animal a reliable PET could be reconstructed due to movement artifacts during the PET. For this reason we decided to only incorporate the CT data of this timepoint.

Selected animals were negative for prior exposure to mycobacteria, as assessed by tuberculin skin testing with Tuberculin PPD RT23 AJV (AJ vaccines, Copenhagen, Denmark) and an Interferon- gamma (IFNγ) ELISPOT assay using Purified Protein Derivative (PPD) from *Mycobacterium bovis*, *Mycobacterium avium* (both Life Technologies NV) or *Mtb* (AJ Vaccines, Copenhagen, Denmark) for *in vitro* recall stimulation of PBMC.

Along the study, the animals were socially housed at biosafety level 3. The monkeys were offered a daily diet consisting of commercial monkey pellets (Sniff, Soest, Germany) supplemented with vegetables and fruit. Homemade and commercially available food and non-food enrichment was provided daily. Drinking water was available ad libitum via a watering system. Animal care staff provided daily visual health checks. The animals were monitored for appetite, general behaviour, and stool consistency. All possible precautions were taken to ensure the welfare and to avoid discomfort to the animals. All experimental interventions (*Mtb* infection, blood samplings and PET-CTs) were performed under anaesthesia (for details see Figure 1). At week 12 post infection (pi) the animals were euthanized with pentobarbital (70 mg/kg), intravenously.

The animals were challenged with *Mtb* strain Erdman K01 (BEI Resource, VA, USA) following a repeated low dose protocol as described before [23]. In short, *Mtb* challenge occurred weekly for eight weeks in a row by endobronchial instillation, targeting the lower left lung lobe with an average dose of one colony forming unit (CFU) in a volume of 3 ml of physiological saline solution. All challenge events were executed in a single session within 2–3 hours from preparing the inoculum from frozen *Mtb* stock, challenging animals in random order. *Mtb* challenge dose was verified for each challenge round by quality control plating.

**
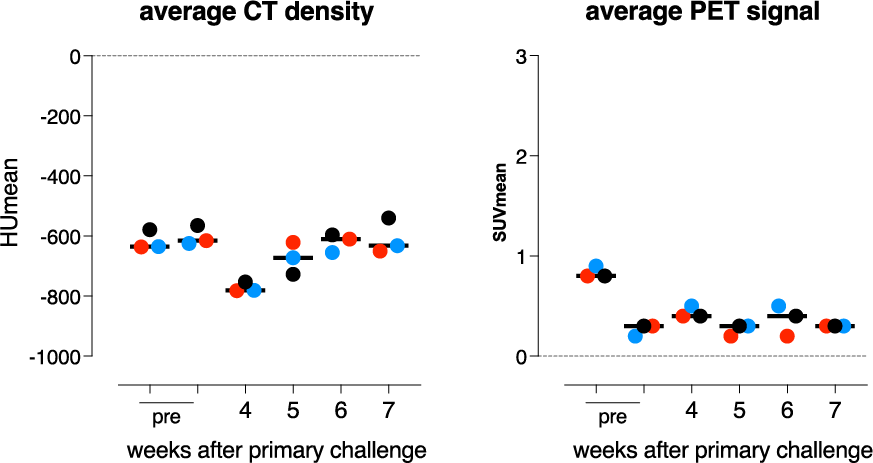
**

**Supplemental Figure 2: Pulmonary PET-CT Background Signals over Time**

Quantitative analysis of the anatomically unaffected lung tissue, based on average CT density represented by HUmean and average PET uptake represented by SUVmean, with the latter dependent on the tracer applied. Both graphs show roughly stable lines; with differences most likely attributable to the image acquisition procedure. The gating applied to CT reconstruction is not always precisely the same part of the breathing cycle. Individual data are plotted, with bars indicating medians.


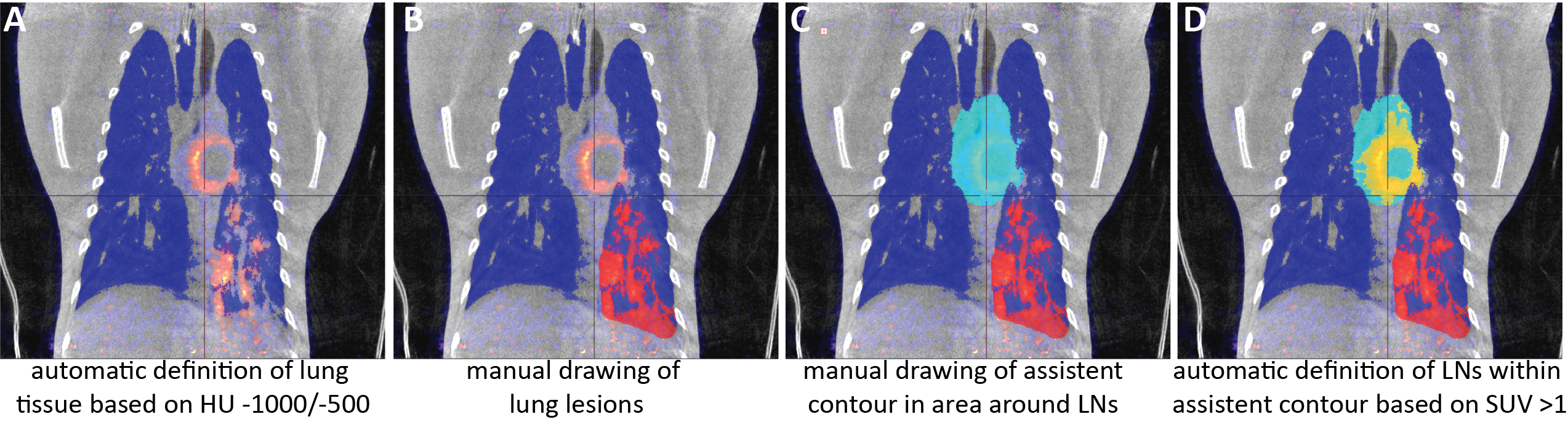


**Supplemental Figure 3: VOI definition**

The procedure to define the different VOIs consists of several steps. First, healthy lung tissue was determined based on a density range (from -1000 to -500 HU) via connected thresholding in Vivoquant. Based on this the PET signals associated with pulmonary lesions were manually delineated by filling the gaps in the healthy lung tissue and afterwards quantified. For the definition of the LNs an assistant contour was generated in the area where the LNs are expected. Followed by a global thresholding with a lower threshold standard uptake value (SUV) of 1.0 to discriminate from the surrounding tissue. This value is based on previous studies and after a visual check in the scans obtained at the last imaging timepoint.
